# Transcriptome and Metabolome Analyses of Short-Term Responses of *Populus talassica × Populus euphratica* to Leaf Damage

**DOI:** 10.3390/ijms26125869

**Published:** 2025-06-19

**Authors:** Mengxu Su, Zhanjiang Han, Ying Liu, Meilin Liu, Lu Guo, Jiaju Wu, Xiaofeng Wu

**Affiliations:** State Key Laboratory Incubation Base for Conservation and Utilization of Bio-Resource in Tarim Basin, College of Life Science and Technology, Tarim University, Alar 843300, China; sumengxu403@163.com (M.S.); liuying836354822@126.com (Y.L.); meilinliu_ruby@163.com (M.L.); guolu15135449921@163.com (L.G.); wujiaju1115@163.com (J.W.); feng18045356866@126.com (X.W.)

**Keywords:** *P. talassica × P. euphratica*, leaf damage, transcriptome, metabolome, short-term responses, MAPK signaling pathway

## Abstract

After being subjected to mechanical damage, plants trigger changes in primary and secondary metabolites to enhance their resistance or defenses. However, there are limited studies on the joint use of transcriptomics and metabolomics in investigating leaf damage-related defense mechanisms and their regulation in woody plants. This study investigated the leaf damage defense mechanisms of *Populus talassica × Populus euphratica* at the molecular level using transcriptome and secondary metabolome analyses. In total, 4078 differentially expressed genes (DEGs; 1207 up-regulated and 2871 down-regulated) and 30 differential secondary metabolites (DSMs; 21 up-regulated and nine down-regulated) were identified from a transcriptome analysis of controls (CK) and CL_75_-treated leaves after 24 h. Plant–pathogen interactions and the MAPK signaling pathway were important defense pathways that synergized in the early stages of leaf damage in *P. talassica × P. euphratica*. There were 44 DEGs enriched in the KEGG pathways that encoded 21 WRKY transcription factors. Flavonoid genes were the most abundant. They were mainly enriched in the flavone and flavonol biosynthesis and flavonoid biosynthesis pathways. Sakuranetin and pinocembrin were most frequently associated with the differential metabolites and may be the main flavonoids involved in responding to leaf damage in *P. talassica × P. euphratica*. This study has far-reaching theoretical and practical significance for understanding the response strategies of *P. talassica × P. euphratica* to leaf damage and for achieving sustainable management and accurate predictions of artificial forests.

## 1. Introduction

As a new cultivar of perennial deciduous trees, *Populus talassica × Populus euphratica* has been popularized in Northwest China [1]. It possesses the excellent characteristics of both *P. talassica* (female parent) and *P. euphratica* (male parent) [2], such as fast growth, straight stems, and salinity–alkalinity and drought tolerance. The growing area and value of *P. talassica × P. euphratica* are easily reduced by *Apocheima cinerarius* infestations, animal damage, and human interference, as determined by a field survey of plantations, farmland, and protective forests in Xinjiang. Therefore, studying the molecular mechanisms following *P. talassica × P. euphratica* leaf damage is beneficial to gain insights into its growth adaptations and defense strategies.

After being subjected to external environmental changes, mechanical damage, or pest-related stress, plants trigger changes in primary and secondary metabolites to enhance their resistance or defenses [3,4]. When the leaves of *Populus simonii × Populus pyramidalis* ‘Opera 8277’ are damaged, H_2_O_2_ accumulates in the undamaged leaves, and the Catalase (CAT) and Superoxide Dismutase (SOD) activities significantly increase [5]. Leaf damage in *Populus deltoides × Populus euramericana* ‘Nanlin 895’ resulted in a significant increase in phenylalanine ammonia-lyase (PAL) activity, not only in its own leaves but also in its neighboring plants. It has been hypothesized that neighboring plants might sense volatile organic compounds released by damaged plants in order to activate their own defense mechanisms before being attacked [6]. After leaf-cutting stimulation, the content of flavonoids in *Pinus tabuliformis* increases, triggering the flavonoid pathway [7].

Transcriptome analysis revealed that after leaf damage to *Jatropha curcas,* the expression of the ribosome-inactivating protein, CURCIN2, was up-regulated in undamaged leaves, along with significant increases in the contents of jasmonic acid and its derivatives [8]. A total of 919 differentially expressed genes (DEGs) were identified in the progeny of *Mimulus guttatus* seedlings after leaf damage [9]. These genes were involved in processes such as cell wall and cell membrane development, stress responses, and secondary metabolic pathways. During leaf-cutting stimulations in *Arabidopsis thaliana*, glutamic acid, which may be a key molecule in the transmission of damage signaling, triggers an increase in calcium ion concentration through glutamate receptor-like ion channels, and this calcium ion concentration increase elicits a defense response in undamaged leaves [10]. In the metabolomics analysis of *Catharanthus roseus* damaged leaves, significant increases in sugar and phenolic acid metabolites were also found in undamaged leaves [11].

However, there are limited studies on the joint use of transcriptomics and metabolomics in investigating leaf damage-related defense mechanisms and their regulation in woody plants. In this study, we used the dominant tree species of ecological forest and timber forest afforestation, *P. talassica × P. euphratica*, and through transcriptome and secondary metabolome analyses of the leaves of *P. talassica × P. euphratica* after a 75% leaf damage treatment (CL_75_), the DEGs and secondary metabolites related to leaf damage were identified. We aimed to analyze the leaf damage-related response mechanisms of the relevant pathways and secondary metabolites in *P. talassica × P. euphratica* and to further understand its growth adaptations and defense strategies.

## 2. Results

### 2.1. Transcriptome Analysis

#### 2.1.1. Quality Control Analysis and Transcriptome Data Annotation

**Figure 1:**
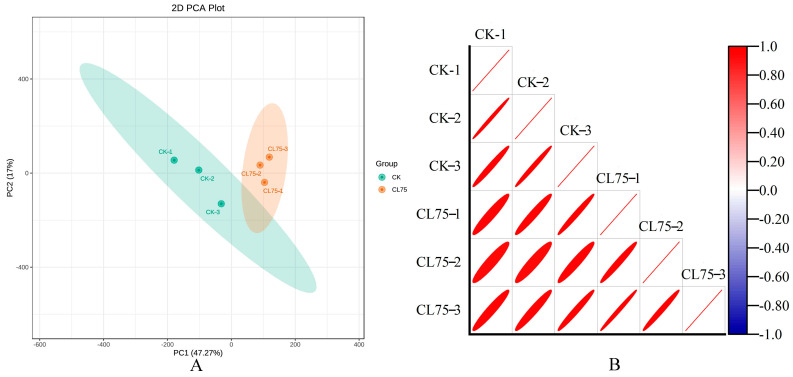
Principal component analysis (**A**) and Pearson correlation heatmap (**B**) of the differentially expressed genes (DEGs).

The sequencing results are shown in Table A1. The raw reads of each sample ranged from 43,678,542 to 53,348,448, and after quality filtering, from 41,507,930 to 50,859,102 clean reads were generated. The total was 265,601,850, which accounted for 95.93% of the raw reads. The clean bases of each sample were distributed in the range of 6.23 to 7.63 G, with Q20 exceeding 97.77%, Q30 exceeding 93.55%, and the average GC content being 43.90%. The reads mapped were in the range of 81.08% to 82.46%. The results indicated that the quality of the transcriptome sequencing data was relatively high. A principal component analysis (PCA) is shown in Figure 1A. The three replicates within the group are close to each other, indicating good repeatability of the sample. There is a clear separation trend between groups, with PC1 determining 47.27% of the variation rate, and PC2 determining 17% of the variation rate. A Pearson correlation heatmap is shown in Figure 1B. The correlation coefficients among the three replicate samples of the same treatment were relatively high, and the groups showed certain differences. Thus, the experimental data could be used for subsequent analyses. Then, the KEGG, GO, NR, Swiss-Prot, Pfam, and KOG databases were used to annotate 20,569, 29,591, 34,366, 26,341, 29,274, and 34,034 single genes, respectively.

#### 2.1.2. Screening and Analysis of DEGs

**Figure 2:**
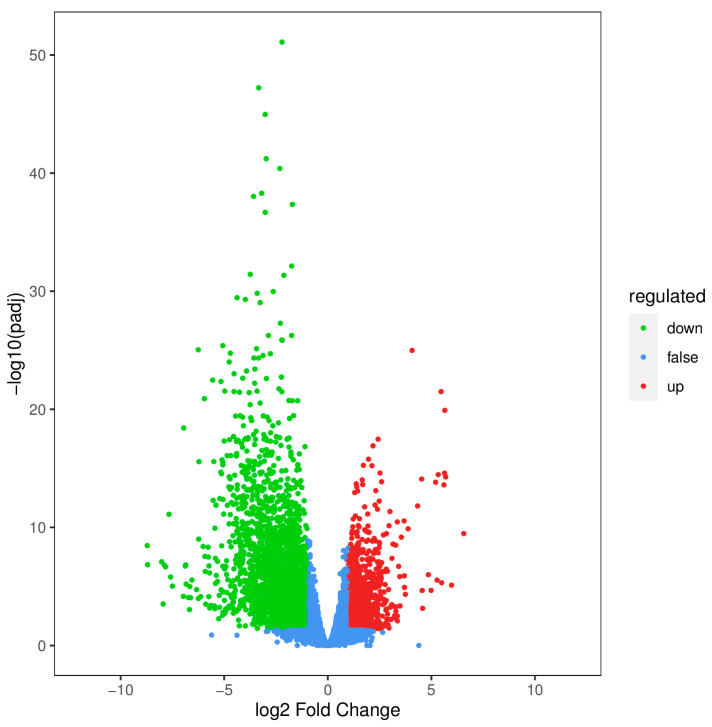
Volcano plot of the DEGs.

Using |log_2_ fold change| ≥ 1 and FDR < 0.05 as screening criteria, a DEG volcano plot was created, as shown in Figure 2. Compared with the CK treatment, 4078 DEGs were identified under the CL_75_ treatment, with 1207 up-regulated and 2871 down-regulated.

#### 2.1.3. GO Enrichment Analysis of DEGs

**Figure 3:**
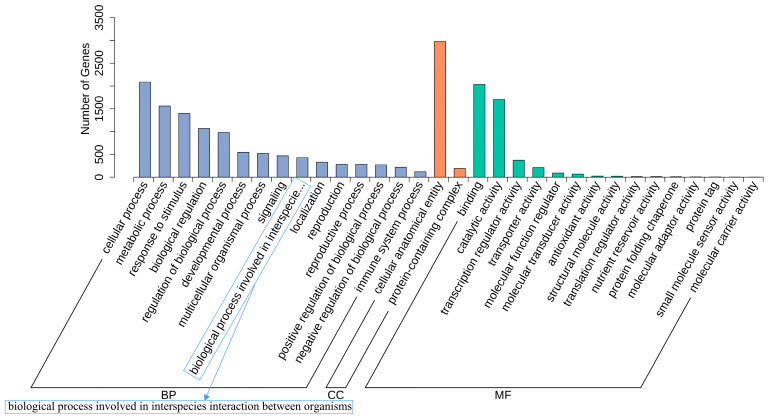
The GO annotation classifications of DEGs.

The DEGs were GO-annotated and analyzed for their expression functions, as shown in Figure 3. The DEGs in the two groups, having 42 different categorized entries, were enriched in three functional categories: Biological Processes, Cellular Components, and Molecular Functions. The DEGs after the leaf damage treatment were highly concentrated in Biological Processes, which mainly included cellular processes, metabolic processes, response to stimuli, biological regulation, and regulation of biological processes. The DEGs annotated in Cellular Components were mainly categorized into cellular anatomical entities and protein-containing complexes. The DEGs annotated in Molecular Functions were mostly focused on binding, catalytic activity, transcription regulator activity, and transporter activity.

#### 2.1.4. KEGG Enrichment Analysis of DEGs

**Figure 4:**
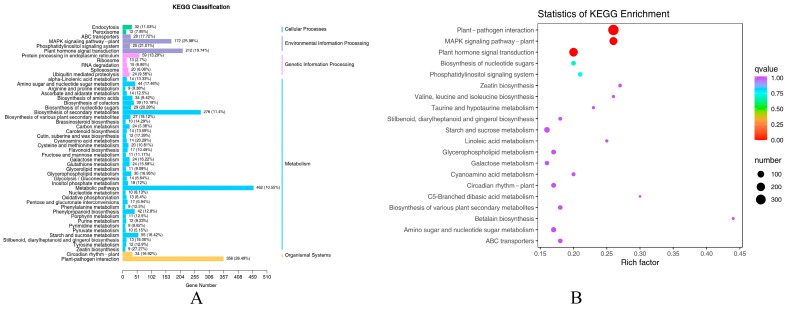
The KEGG pathway classifications and enrichment analysis of DEGs. (**A**) KEGG pathway classification; (**B**) KEGG enrichment analysis. The vertical axis represents the KEGG pathway.

To further analyze the functional differences in gene expression after the leaf damage treatment, a KEGG pathway classification was performed, as shown in Figure 4A. The enriched DEGs were categorized into 50 subclasses in 5 KEGG pathway branches: cellular processes, environmental information processing, genetic information processing, metabolism, and organismal systems. The metabolism pathway had the highest percentage of DEGs, at 58.51%. Among them, metabolic pathways had the highest number, at 462, followed by biosynthesis of secondary metabolites, at 276. There were also 55 and 42 DEGs in starch and sucrose metabolism and phenylpropanoid biosynthesis, respectively. The percentages of DEGs classified in environmental information processing, organismal systems, and genetic information processing were 18.10%, 16.15%, and 5.42%, respectively. Cellular processes had the smallest percentage of DEGs, at 1.82%.

In comparison with the KEGG database, 2748 DEGs involved after leaf injury to *P. talassica × P. euphratica* were enriched in 126 metabolic pathways. The top 20 significantly enriched pathway entries were selected to construct scatter plots, as shown in Figure 4B. The top five up-regulated and down-regulated genes in the three most significantly enriched pathways were functionally annotated using the Nr database, as shown in Table 1. The most enriched pathway was plant–pathogen interaction, with 356 DEGs: 30 up-regulated and 326 down-regulated. The second most enriched pathway was the plant hormone signal transduction pathway, with 212 DEGs: 53 up-regulated and 159 down-regulated. The MAPK signaling pathway—plant had 172 DEGs: 18 up-regulated and 154 down-regulated.

#### 2.1.5. Analysis of the Main KEGG Metabolic Pathways

**Figure 5:**
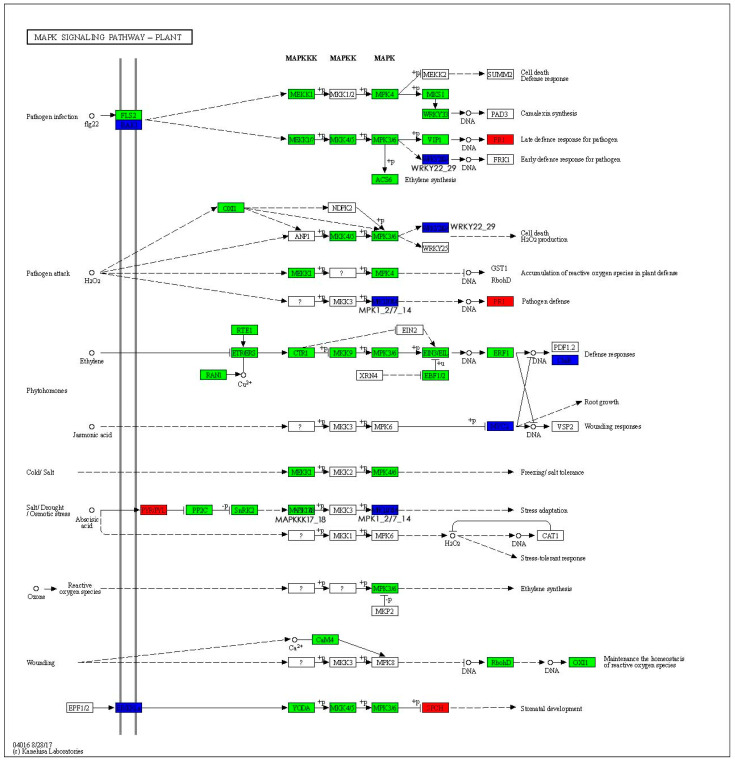
MAPK signaling pathway—plant. The enzymes marked in red boxes are associated with up-regulated genes, while those marked in green boxes are associated with down-regulated genes. The enzymes marked in blue boxes are related to both up-regulated and down-regulated genes.

The MAPK signaling pathway—plant is shown in Figure 5. There is up-regulation of pathogenesis-related protein PR1 (*LOC105111058*), PR4 (*LOC105135405*), the transcription factor bHLH (*LOC105125000*), endochitinase WIN6 (*LOC105140224*, *LOC105140204*), endochitinase WIN8 (*LOC105140947*), etc. Serine/threonine-protein kinase OXI1 (*LOC105125713*), serine/threonine-protein kinase At3g47570 (*LOC105124176*), and mitogen-activated protein kinase MAPK3 (*LOC105113062*) are down-regulated. It focuses on regulating and modulating defense responses, pathogen defense, stomatal development, and maintaining the homeostasis of reactive oxygen species.

#### 2.1.6. Transcription Factor Analysis

In this study, the identified DEGs were mainly annotated to 55 transcription factor families, such as AP2/ERF, WRKY, MYB, and NAC, as shown in Table 2. Among the transcription factors, the AP2/ERF family had the most genes, with 66, including 6 up-regulated and 60 down-regulated, followed by the WRKY gene family (5/40, up-/down-regulated), the MYB family (21/25 up-/down-regulated), the NAC family (3/35 up-/down-regulated), bud differentiation B3 (0/8, up-/down-regulated), calmodulin-binding CAMTA (0/4, up-/down-regulated), and Jumonji (0/3, up-/down-regulated). C2C2-Dof (8), mTERF (5), and SET (5) showed up-regulated expression. It is hypothesized that AP2/ERF, WRKY, MYB, and NAC family genes play important roles in the mechanical damage-related processes in *P. talassica × P. euphratica*.

### 2.2. Secondary Metabolome Analysis

#### 2.2.1. Qualitative and Quantitative Analyses of Metabolites

**Figure 6:**
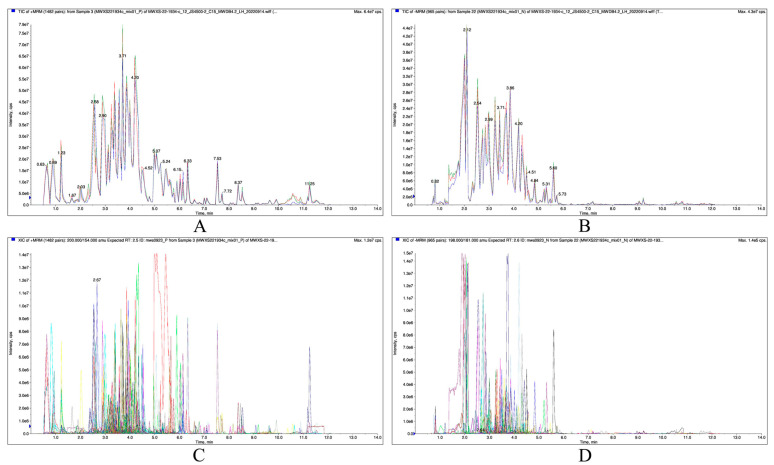
Overlapped total ion current (TIC) of quality control samples (**A**,**B**) and MRM metabolite detection multi-peak diagrams (**C**,**D**). (**A**) TIC of +MRM (1482 pairs): from Sample 3 (MWXS221934c_mix01_P) of MWXS—22—1934—c_12_JS4500—2_C15_MWDB4.2_LH_20220914.wiff (TIC_overlap-P); (**B**) TIC of -MRM (965 pairs): from Sample 22 (MWXS221934c_mix01_N) of MWXS—22—1934—c_12_JS4500—2_C15_MWDB4.2_LH_20220914.wiff (TIC_overlap-P); (**C**) XIC of +MRM (1482 pairs): 200.000/154.000 amu Expected RT: 2.5 ID: mws0923_P from Sample 3 (MWXS221934c_mix01_P) of MWXS-22-1934-c_MRM_detection_of_multimodal_maps-P; (**D**) XIC of -MRM (965 pairs): 198.000/181.000 amu Expected RT: 2.6 ID: mws0923_N from Sample 22 (MWXS221934c_mix01_N) of MWXS-22-1934-c_MRM_detection_of_multimodal_maps-N. (**A**,**B**) Each color chart represents an Extracted Ion Chromatogram chart; (**C**,**D**): Each color represents a detected metabolite. The horizontal axis represents the retention time for metabolite detection, and the vertical axis represents the ion flow intensity for ion detection. “P” stands for positive ion mode, and “N” stands for negative ion mode.

The superposition of the total ion current plots on the mass spectrometry detection of the quality control samples in positive and negative ion modes is shown in Figure 6A,B, respectively. The total ion current curves under the two ion modes were basically similar, with more consistent retention times, good peak shapes, and obvious separations, indicating that the mass spectrometry was good at detecting the same samples at different times, with high signal and instrumental stability. Thus, the signal stability of the mass spectrometry for the same sample at different times was good, and the instrumental stability was strong. Figure 6C,D show the MRM metabolite detection multi-peak diagram in positive and negative ion modes, respectively. In total, 665 secondary metabolites were detected, which were categorized into six types: flavonoids (271, 40.75%), phenolic acids (211, 31.73%), alkaloids (88, 13.23%), lignans and coumarins (56, 8.42%), terpenoids (34, 5.11%), and others (5, 0.75%).

#### 2.2.2. PCA and OPLS-DA

**Figure 7:**
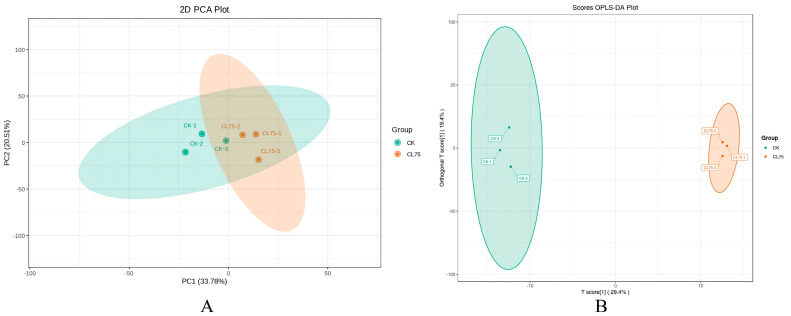
Principal component analysis (**A**) and OPLS-DA Score Plot (**B**).

A PCA of the CK and CL_75_-treated samples revealed the overall metabolic differences between the groups and the degree of variability in the samples within the groups. As can be seen in Figure 7A, the contributions of the first and second principal components were 33.78% and 20.15%, respectively, and the separation of metabolites was more pronounced, indicating a high degree of consistency in the secondary metabolism data within the groups and in differences between the groups. Compared with a PCA, a PLS-DA can maximize the distinction between groups, which is conducive to finding differential secondary metabolites (DSMs). The scores of the two groups were analyzed and plotted according to the OPLS-DA model, as shown in Figure 7B, to further demonstrate the differences between groups. The horizontal coordinate revealed that the control group and the CL_75_-treated group were far apart and obviously different. The vertical coordinate revealed that the sample points within the CL_75_-treated group were closer together and more strongly clustered, indicating that there was less differentiation and more correlation, and the degree of clustering was higher than in the CK group.

#### 2.2.3. Screening DSMs

As shown in Table 3, based on the OPLS-DA, 30 DSMs were screened. Twenty-one had up-regulated expression levels, and nine had down-regulated expression levels. They were selected using the criteria fold change ≥ 2 or ≤ 0.5 and VIP ≥ 1, and they included flavonoids, terpenoids, phenolic acids, alkaloids, lignans and coumarins, and others. Further screening (*P*-value < 0.05) yielded 13 significant DSMs, 11 with up-regulated and 2 with down-regulated expression levels. In the order of their multiplicity of difference, 10 flavonoids, rhamnetin, 3,5,3′,4′-tetrahydroxy-7-methoxyflavone, 3-O-methylquercetin, 3-O-acetylpinobanksin, chrysoeriol, 5,7,4′-trihydroxy-3′-methoxyflavone, nepetin (5,7,3′,4′-tetrahydroxy-6-methoxyflavone), acacetin, genkwanin (apigenin 7-methyl ether), prunetin (5,4′-dihydroxy-7-methoxyisoflavone), 6,7,8-tetrahydroxy-5-methoxyflavone, and chrysin, and one terpenoid, 3,19-dihydroxyurs-12-en-28-oic acid (pomolic acid), that were significantly increased. In total, two lignans and coumarins, trans-1,2-dihydrodehydroguaiaretic acid and dehydrodiisoeugenol, were significantly decreased.

#### 2.2.4. Correlation Analysis of DSMs

**Figure 8:**
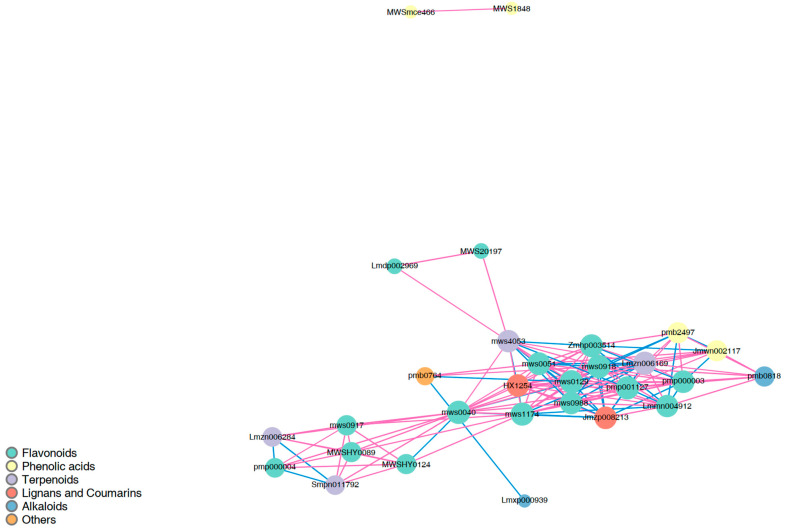
Correlation network diagram of DSMs. The dots in the graph represent different DSMs, the red lines represent positive correlations, and the blue lines represent negative correlations. The thickness of the line represents the absolute value of the Pearson correlation coefficient r, and the thicker the line, the larger |r|. By default, the differential secondary metabolite pairs are plotted with |r| > 0.8 and *p* < 0.05.

The DSM correlation network diagram is shown in Figure 8. Among the flavonoids, *MWSHY0089* (sakuranetin) and *MWSHY0124* (pinocembrin) caused changes in the contents of 29 and 25 metabolites, respectively.

#### 2.2.5. KEGG Enrichment Analysis of DSMs

**Figure 9:**
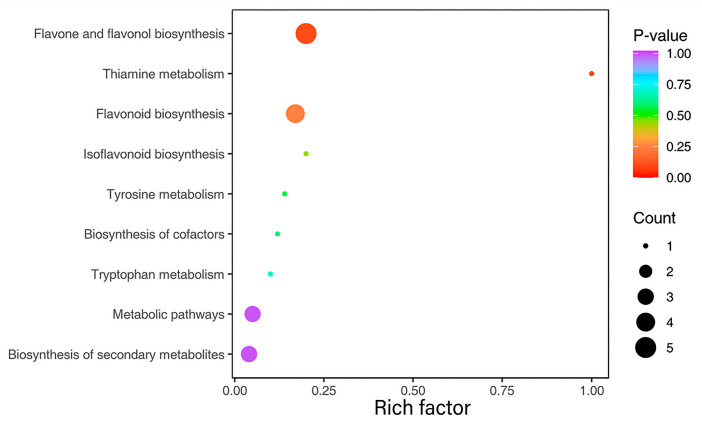
KEGG enrichment analysis of DSMs. The vertical axis represents the KEGG pathway.

The DSMs between the two groups were subjected to KEGG annotation, and they were involved in nine metabolic pathways, as shown in Figure 9. The top three pathways that were significantly enriched were ko00944 (flavone and flavonol biosynthesis), ko00730 (thiamine metabolism), and ko00941 (flavonoid biosynthesis).

### 2.3. Integration Analyses of Transcriptome and Secondary Metabolome

#### 2.3.1. KEGG Enrichment Analysis

**Figure 10:**
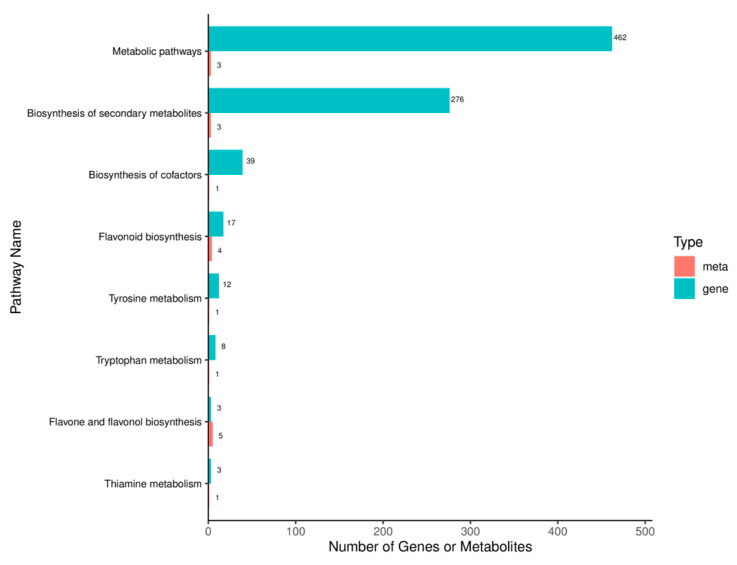
KEGG pathway enrichment with both DEGs and DSMs. The horizontal axis represents the number of differentially expressed metabolites and genes enriched in this pathway, the vertical axis represents the KEGG pathway name, and the red and green bars represent the metabolome and transcriptome, respectively.

The KEGG pathways that were co-enriched in the transcriptome and the secondary metabolome were determined, as were the numbers of DEGs and DSMs shared in each pathway, as shown in Figure 10. The highest number of DEGs, 462, was enriched in the metabolic pathways, followed by biosynthesis of secondary metabolites, with 276 DEGs. Biosynthesis of cofactors was third in the ranking, with 39 DEGs. DSMs were enriched in flavone and flavonol biosynthesis, with five DSMs, flavonoid biosynthesis, with four, and metabolic pathways and biosynthesis of secondary metabolites, with three each.

#### 2.3.2. Correlation Cluster Analysis

**Figure 11:**
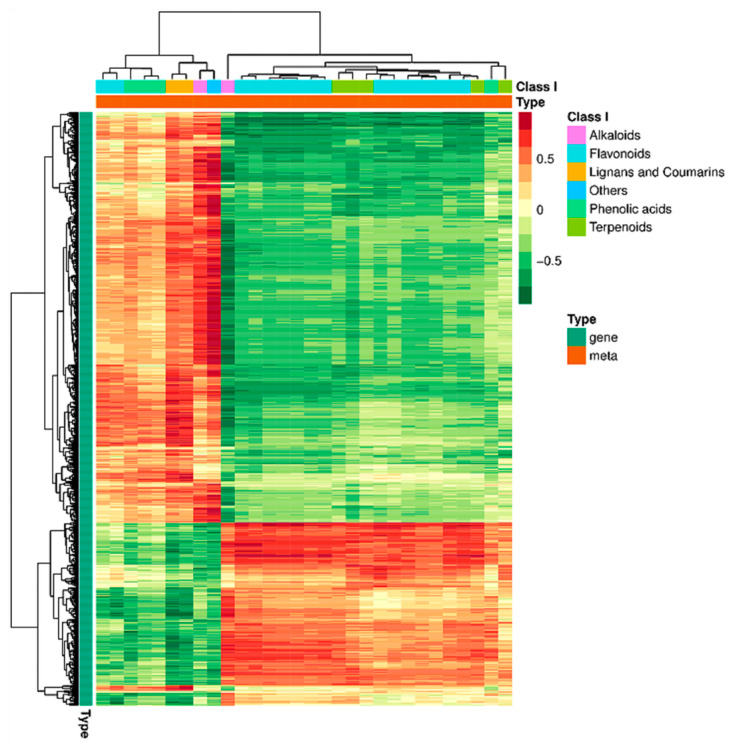
Heatmap of correlation clustering between DEGs and DSMs. Behavioral genes are listed as metabolites, with red representing a positive correlation and green representing a negative correlation.

All the correlations between DEGs and DSMs were calculated, and a correlation clustering heatmap was constructed (Figure 11). The DEGs between the two groups showed higher negative correlations with flavonoids and terpenoids and higher positive correlations with lignans and coumarins and phenolic acids.

#### 2.3.3. Typical Correlation Analysis

**Figure 12:**
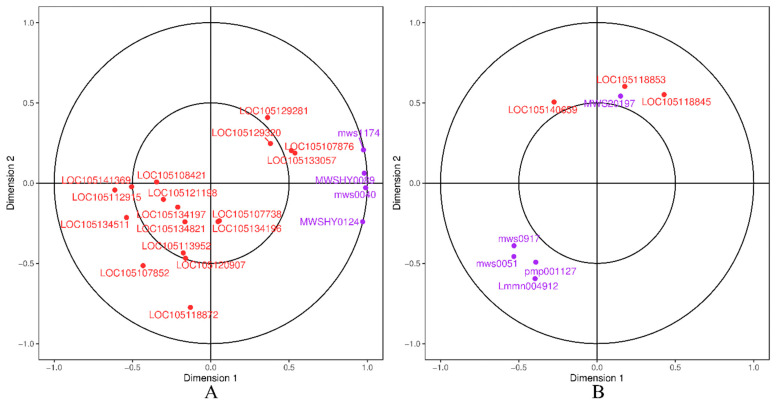
Canonical correlation analysis of DEGs and DSMs. (**A**): ko00941 (flavonoid biosynthesis); (**B**): ko00944 (flavone and flavonol biosynthesis). Purple represents metabolites, while red represents genes.

A typical correlation analysis of DEGs and DSMs in the ko00941 (flavonoid biosynthesis) and ko00944 (flavone and flavonol biosynthesis) pathways is shown in Figure 12. The flavonoids 3-O-acetylpinobanksin (*mws1174*) and sakuranetin (*MWSHY0059*), which were highly correlated with the genes *LOC105107876* and *LOC105133057*, respectively, were up-regulated and expressed (Figure 12A). However, the flavonoids pinocembrin (*MWSHY0124*) and chrysin (*mws0040*) were weakly correlated with other genes. The flavonoid quercitrin (*MWS20197*), which has a higher correlation with the genes *LOC105118853* and *LOC105118845*, was down-regulated in expression (Figure 12B).

#### 2.3.4. Summary Table for DEGs, DSMs, and Pathways

Based on the above results, it can be concluded that the leaf damage of *P. talassica × P. euphratica* involves several key transcription factors, such as AP2/ERF, WRKY, MYB, and NAC, and some important genes and metabolites, as well as metabolic pathways, as shown in Table 4.

## 3. Discussion

### 3.1. Transcriptome Analysis of P. talassica × P. euphratica in Response to Leaf Damage

Compared with the CK group, 4078 DEGs were identified in the CL_75_-treated group: 1207 were up-regulated and 2871 were down-regulated. These DEGs were most significantly enriched in the plant–pathogen interaction pathway. It was speculated that after leaf damage to *P. talassica × P. euphratica*, damage-related signals were transmitted into the nucleus, which activated or repressed the transcription of related genes through transcription factors. These factors then up-regulated the expression levels of calcium-binding protein calmodulin-like 23 (*LOC105132625*) and a leucine-rich repeat receptor-like Ser/Thr-protein kinase (*LOC105138044*, *LOC105129033*), which may have induced a hypersensitive response that regulated stomatal opening and closing and cell wall strengthening, resulting in a rapid response to leaf damage. The calmodulin-like gene family is a unique calcium sensor in plants and is involved in signal transduction for growth and development, biotic and abiotic stress responses, and hormone actions in higher plants [12]. Leucine-rich repeat receptor-like kinases are the largest class of receptor-like protein kinases [13], and they act as receptors for signal recognition and participate in signal transduction. They also have important regulatory roles in plant responses to adversity, in growth and development, and in signal transduction [14]. It has been hypothesized that *P. talassica × P. euphratica* can synergistically resist stress by regulating binding proteins and protein kinases during leaf damage treatments [15]. As shown in Table 2, the expression levels of some calcium-binding proteins and receptor-like protein kinases were reduced after leaf damage to *P. talassica × P. euphratica*. The decrease in the expression of these substances, to a certain extent, may lead to the blocking of signaling and metabolic pathways in the tree, making it difficult for the tree to respond to external stimuli in a timely manner. This may negatively activate other regulatory genes that jointly help the tree resist damage-related stress.

The mitogen-activated protein kinase (MAPK) cascade pathway, which consists of the MAPKKK-MAPKK-MAPK tertiary protein kinase system, is a signaling pathway commonly existing in eukaryotes that plays a key role in responding to biotic or abiotic stresses, as well as hormonal and developmental signals [16]. Research has revealed that RAF14 and RAF79 may act as key factors in the regulation of nitrogen metabolism by MAPK [17]. It also plays an important function in the early stages of damage signaling to activate downstream defense responses [18,19]. An analysis of the MAPK signaling pathway in plants revealed that most of the key enzymes of the tertiary kinase module have down-regulated expression levels, which induces the up-regulated expression of disease process-related proteins to stimulate stomatal development and responses to pathogens. Moreover, the down-regulated expression levels of the respiratory burst oxidase and Ser/Thr protein kinase disrupt reactive oxygen species homeostasis in vivo. These two pathways were significantly enriched in *P. talassica × P. euphratica* in response to leaf damage. It has been hypothesized that leaf damage serves as an injury signal, activating pathways associated with resistance to pathogen invasion, which, in turn, exhibit physiological resistance to pathogen infestations that may result from wounding [20,21]. Thus, plant–pathogen interactions and the MAPK signaling pathway in plants play important roles as defense-related pathways that synergize in the early stages of leaf damage to *P. talassica × P. euphratica*. In addition, in the phytohormone signaling pathway, the abscisic acid receptor PLY4-like, the scarecrow-like protein related to hormone signaling transduction [22], and the transcription activator GLK1-like [23], which is associated with leaf phenotypes, showed up-regulated expression levels. This up-regulation may activate or silence related pathways, thereby regulating hormonal responses and leaf changes, which assist *P. talassica × P. euphratica* in responding to leaf damage.

### 3.2. Transcription Factor Analysis of P. talassica × P. euphratica in Response to Leaf Damage

When plants are subjected to mechanical injury, they may regulate the expression of defense-related genes and exercise their functions through a complex signaling series, thereby maximizing adaptation to the environment. In this process, transcription factors play crucial roles as transcription initiation switches. Among them, WRKY transcription factors, which regulate the expression of defense-related genes in plants, play important roles in disease defense, mechanical damage response, and senescence [24,25]. These processes are induced by external factors, which, in turn, positively or negatively regulate the expression of target genes. A screened *WIZZ* gene encoding a WRKY transcription factor is transiently activated and rapidly accumulates in damaged and undamaged leaves of *Nicotiana tabacum* [26]. The overexpression of *WRKY44* from *Hibiscus cannabinus* in *A. thaliana* improves its salt tolerance by positively regulating the expression of the Na^+^/H^+^ anti-transporter protein gene *SOS1* [27]. The overexpression of *NtWRKY65* significantly reduces the Na^+^ content and increases the K^+^/Na^+^ ratio, which is a key regulator of salt tolerance in *N. tabacum* [28]. *PoWRKY69* is overexpressed in *Paeonia ostii*, enhancing drought tolerance [29].

In this study, 44 DEGs enriched in the KEGG pathway were annotated to 21 WRKY transcription factors using the Nr database. In the plant–pathogen interaction pathway, the genes annotated as transcription factors *WRKY7* (*LOC105129145*), *WRKY65* (*LOC105135969*), *WRKY69* (*LOC105137987*), and *WRKY44* (*LOC105110708*, *LOC105142478*) exhibited up-regulated expression levels, which may positively regulate the induced defense-related genes involved in the response to leaf damage in *P. talassica × P. euphratica*. In *A. thaliana*, ABA-related mutants induce the down-regulated expression of *AtWRKY46*, which regulates ABA signaling and growth hormone homeostasis, thereby contributing to the feed-forward prevention of osmotic or salt stress-dependent lateral root inhibition [30]. Thus, the down-regulated expression of genes annotated to *WRKY46* in the MAPK signaling pathway may lead to a large H_2_O_2_ accumulation in plants through negative regulation, thereby causing an early defense response, whereas osmotic stress induces the up-regulated expression of the ABA receptor. This up-regulation accelerates the senescence of the damaged parts, thereby insulating the areas from the threat of the external environment. In this manner, senescence acts as a defense. This is similar to the findings in *Zoysia japonica* [31] in response to leaf damage.

### 3.3. Secondary Metabolome Analysis of P. talassica × P. euphratica in Response to Leaf Damage

Secondary metabolites play important roles in plant defense against stress and damage. The flavonoid contents of plants significantly increase after mechanical damage, insect damage, and jasmonic acid spraying [7,32]. In this study, 665 secondary metabolites from six major classes were detected using a targeted secondary metabolome analysis. They contained 271 flavonoids and 211 phenolic acids. A total of 30 DSMs, mainly including flavonoids, flavonols, isoflavones, and dihydroflavonoids, were identified, and they were mainly enriched in the pathways of flavonoid and flavonol biosynthesis and flavonoid biosynthesis. Therefore, flavonoids play important roles in responses to leaf damage in *P. talassica × P. euphratica*. More DSMs showed increased expression than decreased expression, suggesting that the synthesis and accumulation of secondary metabolites were promoted in response to leaf damage in *P. talassica × P. euphratica*. According to the DSM correlation network diagram, sakuranetin (*MWSHY0089*) and pinocembrin (*MWSHY0124*) were associated with the most metabolites, causing changes in the contents of 29 and 25 metabolites, respectively. Thus, it was hypothesized that changes in flavonoid contents following leaf damage in *P. talassica × P. euphratica* initiate a complex osmoregulatory network to improve the ability of *P. talassica × P. euphratica* to withstand leaf damage. Flavonoids help to improve plant stress tolerance. For example, flavonoids are involved in the response to salt stress by scavenging free radicals and increasing the antioxidant capacity of *Sophora alopecuroides* [33]. Sakuranetin is a flavonoid phytochemical in *Oryza sativa* that is exclusively resistant to rice blast and is effective against pathogenic bacteria [34]. *Ginkgo biloba* shows a significant increase in total flavonoid content and accumulations of sakuranetin, hesperidin, and cinnamic acid under drought stress conditions, and the flavonoid biosynthesis pathway plays a crucial role in *G. biloba* in response to drought stress [35]. Here, the leaf damage treatment induced a rapid increase in the flavonoids in the leaves of *P. talassica × P. euphratica* but had no significant effects on other secondary metabolites. This was probably because the mechanical damage caused by the leaf damage treatment was more of a physical stimulus for *P. talassica × P. euphratica*, as opposed to a chemical stimulus, like those from diseases and insects. There was a delay in the induction of secondary metabolites, such as hormones and alkaloids.

## 4. Materials and Methods

### 4.1. Site Description

The experimental site was located in the seedling base of the 10th Regiment of the 1st Division, Alar, Xinjiang (81°18′08″ E, 40°36′13″ N, with an altitude of 1014 m), which has an extreme continental, arid desert climate in a warm temperate zone. The average annual temperature, sunshine, and precipitation are 12.1 °C, 2568.5 h, and 54.1 mm, respectively. The basic physicochemical properties of the tested soil are shown in Table A2.

### 4.2. Plant Material and Experimental Treatments

**Figure 13:**
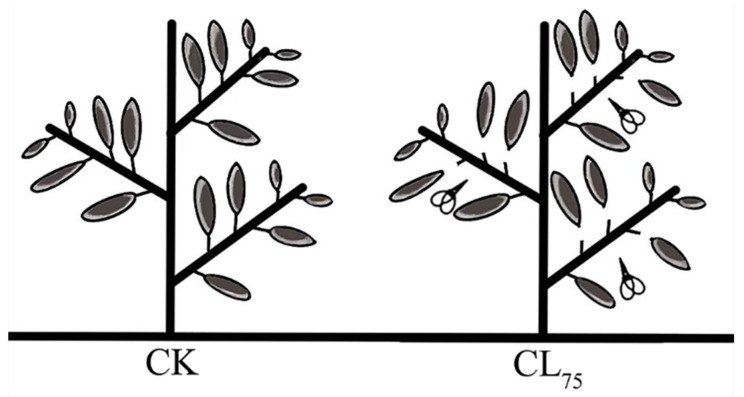
Diagram of the leaf damage treatment. CK: control; CL_75_: starting from the bottom upwards, three out of every four leaves were pruned, resulting in the amount of leaves pruned accounting for 75% of the total leaves on the whole plant.

Well-grown, uniform, and pest-free seedlings of *P. talassica × P. euphratica* were selected as test materials. For leaf cutting 75% (CL_75_), starting from the plant bottom, three out of every four leaves were cut; only fully expanded leaves were cut without damaging the terminal buds (Figure 13). Undamaged healthy plants were used as controls (CKs). Fresh, undamaged leaves were collected 24 h after treatment, mixed thoroughly, and then quickly stored in an ultra-low-temperature refrigerator at −80 °C after treatment with liquid nitrogen. Each treatment had three replicates, with three seedlings per replicate. High-throughput transcriptome sequencing and secondary metabolome analyses were performed by Metware Biotechnology Co., Ltd., Wuhan, China.

### 4.3. Transcriptome Analysis

Using Illumina sequencing technology, clean reads were compared with the reference genome of *P. euphratica* (https://ftp.ncbi.nlm.nih.gov/genomes/all/GCF/000/495/115/GCF_000495115.1_PopEup_1.0/ (accessed on 26 July 2023)) using HISAT2 (Version 2.1.0) (https://daehwankimlab.github.io/hisat2/ (accessed on 8 June 2017)) to obtain their positional information on the reference genome or gene [36]. And StringTie (Version 1.3.3) (https://ccb.jhu.edu/software/stringtie/ (accessed on 15 February 2017)) was used to perform quantitative expression analysis on each sample gene [37]. A differential expression analysis between sample groups was carried out using DESeq2 [38,39], and the Benjamini–Hochberg method was used to correct for multiple hypothesis testing probabilities (*P*-values) to obtain the final FDR value. The criteria |log_2_ (fold change)| ≥ 1 and FDR < 0.05 were used to screen DEGs. The screened DEGs were annotated against the KEGG, GO, KOG, NR, Swiss-Prot, and Pfam databases to obtain the protein functional annotation information corresponding to the DEGs. The DEGs were then analyzed for GO functional enrichment and KEGG metabolic pathway enrichment.

### 4.4. Secondary Metabolome Analysis

Qualitative and quantitative analyses were conducted by leveraging the Metware database (Metware Biotechnology Co. Ltd. Wuhan, China) and the multiple reaction monitoring mode of triple-quadrupole mass spectrometry. DSMs were screened using the criteria VIP ≥ 1 and fold change ≥ 2 or fold change ≤ 0.5. The metabolites were annotated using the KEGG database [40], and pathway classification and enrichment analyses were performed using the KEGG annotations of the DSMs. Afterward, the associations of DEGs with DSMs were analyzed.

## 5. Conclusions

In this study, transcriptome and secondary metabolome analyses of CK and CL_75_-treated *P. talassica × P. euphratica* leaves were performed, and 4078 DEGs (1207 up-regulated and 2871 down-regulated) and 30 DSMs (21 up-regulated and nine down-regulated) were identified. The KEGG enrichment analysis revealed important regulatory pathways, such as the plant–pathogen interaction, MAPK signaling, and plant hormone signaling transduction, and the roles of potential WRKY transcription factors in response to leaf damage were preliminarily analyzed. There were 44 DEGs enriched in the KEGG pathways that encoded 21 WRKY transcription factors. Among them, the genes annotated as transcription factors *WRKY7* (*LOC105129145*), *WRKY65* (*LOC105135969*), *WRKY69* (*LOC105137987*), and *WRKY44* (*LOC105110708*, *LOC105142478*) exhibited up-regulated expression levels, which may play crucial roles in the response of *P. talassica × P. euphratica* to damage stress. Flavonoids were the most abundant and up-regulated among the DSMs. They were mainly enriched in the flavone and flavonol biosynthesis and flavonoid biosynthesis metabolism pathways, and sakuranetin and pinocembrin were most frequently associated with the differential metabolites, indicating that they may be the main flavonoids involved in the response to leaf damage in *P. talassica × P. euphratica*. This study has far-reaching theoretical and practical significance for understanding the response strategies of *P. talassica × P. euphratica* to leaf damage and for achieving the sustainable management and accurate predictions of artificial forests. The results provide a theoretical basis for further research on the molecular mechanisms of damage tolerance in *P. talassica × P. euphratica*. In the future, the functions of the relevant genes will be validated, and the regulatory mechanism of WRKY transcription factors will be explored to analyze the regulatory mechanisms of *P. talassica × P. euphratica* in response to leaf damage.

## Figures and Tables

**Table 1:** Related DEGs in core pathways and their predicted functional annotations.

Pathway	Gene ID	log_2_ Fold Change	Nr Functional Annotation
plant–pathogen interaction	LOC105111058	2.39	PREDICTED: STS14 protein-like [*P. euphratica*]
	LOC105127365	2.35	PREDICTED: ethylene-responsive transcription factor CRF1 [*P. euphratica*]
	LOC105138044	2.11	PREDICTED: probable LRR receptor-like serine/threonine-protein kinase At1g67720 [*P. euphratica*]
	LOC105132085	1.68	PREDICTED: elongation factor Tu, mitochondrial [*P. euphratica*]
	LOC105138760	1.58	PREDICTED: protein STRUBBELIG-RECEPTOR FAMILY 3 isoform X1 [*P. euphratica*]
	LOC105124176	−5.13	PREDICTED: probable LRR receptor-like serine/threonine-protein kinase At3g47570 [*P. euphratica*]
	LOC105124276	−5.16	PREDICTED: probable WRKY transcription factor 41 [*P. euphratica*]
	LOC105112185	−5.43	PREDICTED: receptor-like cytosolic serine/threonine-protein kinase RBK2 isoform X1 [*P. euphratica*]
	LOC105110565	−6.22	PREDICTED: calcium-binding protein CML38 [*P. euphratica*]
	LOC105135772	−7.59	PREDICTED: 3-ketoacyl-CoA synthase 1-like [*P. euphratica*]
plant hormone signal transduction	LOC105141486	4.56	PREDICTED: hypothetical protein POPTR_009G126000v3 [*P. trichocarpa*]
	LOC105121906	3.88	PREDICTED: abscisic acid receptor PYL4-like [*P. euphratica*]
	LOC105136244	2.47	PREDICTED: probable carboxylesterase 8 [*P. euphratica*]
	LOC105139037	2.43	PREDICTED: transcription activator GLK1-like isoform X1 [*P. euphratica*]
	LOC105119527	2.43	PREDICTED: scarecrow-like protein 4 [*P. euphratica*]
	LOC105121293	−4.68	PREDICTED: probable xyloglucan endotransglucosylase/hydrolase protein 23 [*P. euphratica*]
	LOC105112185	−5.43	PREDICTED: receptor-like cytosolic serine/threonine-protein kinase RBK2 isoform X1 [*P. euphratica*]
	LOC105129715	−5.70	PREDICTED: leucine-rich repeat receptor protein kinase EXS-like [*P. euphratica*]
	LOC105121294	−5.74	PREDICTED: probable xyloglucan endotransglucosylase/hydrolase protein 23 [*P. euphratica*]
	LOC105141318	−5.93	PREDICTED: probably inactive leucine-rich repeat receptor-like protein kinase IMK2 [*P. euphratica*]
MAPK signaling pathway—plant	LOC105140947	5.65	PREDICTED: endochitinase WIN8 isoform X1 [*P. euphratica*]
	LOC105121906	3.88	PREDICTED: abscisic acid receptor PYL4-like [*P. euphratica*]
	LOC105125000	3.36	PREDICTED: transcription factor bHLH94-like [*P. euphratica*]
	LOC105140224	3.00	PREDICTED: acidic endochitinase WIN6 [*P. euphratica*]
	LOC105140204	2.66	PREDICTED: acidic endochitinase WIN6-like [*P. euphratica*]
	LOC105131648	−4.71	PREDICTED: receptor-like protein kinase isoform X1 [*P. euphratica*]
	LOC105131478	−5.07	PREDICTED: probable WRKY transcription factor 33 [*P. euphratica*]
	LOC105124176	−5.13	PREDICTED: probable LRR receptor-like serine/threonine-protein kinase At3g47570 [*P. euphratica*]
	LOC105124276	−5.16	PREDICTED: probable WRKY transcription factor 41 [*P. euphratica*]
	LOC105112185	−5.43	PREDICTED: receptor-like cytosolic serine/threonine-protein kinase RBK2 isoform X1 [*P. euphratica*]

**Table 2:** Classification of the annotated transcription factor families.

Transcription Factor	Number of DEGs	Up	Down	Transcription Factor	Number of DEGs	Up	Down
Alfin-like	1	1	0	IWS1	1	0	1
AP2/ERF-AP2	2	2	0	Jumonji	4	0	4
AP2/ERF-ERF	62	4	58	LOB	7	3	4
AP2/ERF-RAV	2	0	2	MADS-M-type	1	0	1
AUX/IAA	6	3	3	MADS-MIKC	1	0	1
B3	8	0	8	MBF1	1	0	1
B3-ARF	3	2	1	mTERF	5	5	0
bHLH	26	7	19	MYB	33	12	21
bZIP	9	5	4	MYB-related	13	9	4
C2C2-CO-like	8	6	2	NAC	38	3	35
C2C2-Dof	8	8	0	NF-YA	3	2	1
C2C2-GATA	7	1	6	NF-YB	2	2	0
C2H2	17	6	11	NF-YC	2	0	2
C3H	12	3	9	Others	14	4	10
CAMTA	4	0	4	Pseudo ARR-B	3	3	0
CSD	1	1	0	SBP	5	2	3
DBB	2	1	1	SET	4	4	0
EIL	1	0	1	SNF2	3	0	3
FAR1	1	1	0	SWI/SNF-BAF60b	1	0	1
GARP-ARR-B	2	1	1	TAZ	1	1	0
GARP-G2-like	8	6	2	TCP	3	2	1
GNAT	4	1	3	Tify	7	1	6
GRAS	22	9	13	TRAF	3	0	3
GRF	1	1	0	Trihelix	4	1	3
HB-BELL	1	0	1	TUB	1	0	1
HB-HD-ZIP	5	3	2	WRKY	45	5	40
HB-WOX	1	0	1	zf-HD	4	3	1
HSF	7	2	5	/			

**Table 3:** Statistics of differential secondary metabolites (DSMs).

Index	Class I	Compounds	Formula	Type
MWSHY0089	Flavonoids	5,4′-Dihydroxy-7-methoxyflavanone (Sakuranetin)	C_16_H_14_O_5_	up
MWSHY0124	Flavonoids	Pinocembrin (Dihydrochrysin)	C_15_H_12_O_4_	up
Zmhp003514	Flavonoids	6,7,8-Tetrahydroxy-5-methoxyflavone	C_16_H_12_O_6_	up
mws0988	Flavonoids	Rhamnetin; 3,5,3′,4′-Tetrahydroxy-7-Methoxyflavone	C_16_H_12_O_7_	up
mws0918	Flavonoids	Prunetin (5,4′-Dihydroxy-7-methoxyisoflavone)	C_16_H_12_O_5_	up
mws1174	Flavonoids	3-O-Acetylpinobanksin	C_17_H_14_O_6_	up
Lmmn004912	Flavonoids	3-O-Methylquercetin	C_16_H_12_O_7_	up
mws0129	Flavonoids	Genkwanin (Apigenin 7-methyl ether)	C_16_H_12_O_5_	up
mws0040	Flavonoids	Chrysin	C_15_H_10_O_4_	up
mws0051	Flavonoids	Acacetin	C_16_H_12_O_5_	up
pmp001127	Flavonoids	Chrysoeriol; 5,7,4′-Trihydroxy-3′-Methoxyflavone	C_16_H_12_O_6_	up
pmp000003	Flavonoids	Nepetin (5,7,3′,4′-Tetrahydroxy-6-methoxyflavone)	C_16_H_12_O_7_	up
mws0917	Flavonoids	3,7-Di-O-methylquercetin	C_17_H_14_O_7_	up
pmp000004	Flavonoids	4′,5,7-Trihydroxy-3′,6-dimethoxyflavone (Jaceosidin)	C_17_H_14_O_7_	up
MWS20197	Flavonoids	Quercetin-3-O-rhamnoside (Quercitrin)	C_21_H_20_O_11_	down
Lmdp002969	Flavonoids	Myricetin-3-O-galactoside	C_21_H_20_O_13_	down
mws4053	Terpenoids	3-Hydroxyurs-12-en-28-oic acid (Ursolic acid)	C_30_H_48_O_3_	up
Smpn011792	Terpenoids	2,3-Dihydroxy-12-ursen-28-oic acid	C_30_H_48_O_4_	up
Lmzn006169	Terpenoids	3,19-Dihydroxyurs-12-en-28-oic acid (Pomolic acid)	C_30_H_48_O_4_	up
Wbmn009702	Terpenoids	2,3,23-Trihydroxyurs-12-en-28-oic acid	C_30_H_48_O_5_	up
Lmzn006284	Terpenoids	2-Hydroxyursolic acid	C_30_H_48_O_4_	up
pmb2497	Phenolic acids	4-Hydroxy-3-methoxymandelate	C_9_H_10_O_5_	up
MWS1848	Phenolic acids	Phenyl acetate	C_8_H_8_O_2_	down
Jmwn002117	Phenolic acids	2-(3,4-dihydroxyphenyl)ethanediol 1-O-β-D-glucopyranoside	C_14_H_20_O_9_	down
MWSmce466	Phenolic acids	4-Hydroxyacetophenone	C_8_H_8_O_2_	down
pmb0818	Alkaloids	Methoxyindoleacetic acid	C_11_H_11_NO_3_	up
Lmxp000939	Alkaloids	Zarzissine	C_5_H_5_N_5_	down
HX1254	Lignans and Coumarins	trans-1,2-dihydrodehydroguaiaretic acid	C_20_H_22_O_4_	down
Jmzp008213	Lignans and Coumarins	Dehydrodiisoeugenol	C_20_H_22_O_4_	down
pmb0764	Others	4-Methyl-5-thiazoleethanol	C_6_H_9_NOS	down

**Table 4:** DEGs, DSMs, and pathways for leaf damage.

	Leaf Damage
transcription factors	AP2/ERF, WRKY, MYB, NAC
DEGs	*LOC105111058, LOC105125000, LOC105125713, LOC105132625, LOC105138044, LOC105129033, LOC105129145, LOC105135969, LOC105110708, LOC105142478*
DSMs	flavonoids, terpenoids, phenolic acids
metabolic pathways	flavone and flavonol biosynthesis, thiamine metabolism, flavonoid biosynthesis

## Data Availability

The original contributions presented in this study can be found in online repositories. The sequenced raw reads (SRA) were deposited in the NCBI Sequence Read Archive. Accession number: PRJNA1215557; TaxID: 2929483 (https://dataview.ncbi.nlm.nih.gov/object/PRJNA1215557) (accessed on 2 June 2025).

## Abbreviations

The following abbreviations are used in this manuscript:

ABA	Abscisic Acid
CAT	Catalase
FDR	False Discovery Rate
GO	Gene Ontology
KEGG	Kyoto Encyclopedia of Genes and Genomes
KOG	Clusters of Orthologous Groups for Eukaryotic Complete Genomes
MRM	Multiple Reaction Monitoring
NR	Non-Redundant Protein Sequence Database
OPLS-DA	Orthogonal Partial Least Squares Discriminant Analysis
PAL	Phenylalanine ammonia-lyase
SOD	Superoxide Dismutase
VIP	Variable Importance in Projection

## Appendix A

**Table A1:** Quality-related statistics of the sequencing data.

Sample	Raw Reads	Clean Reads	Reads Mapped (%)	Clean Base (G)	Error Rate (%)	Q20 (%)	Q30 (%)	GC Content (%)
CK-1	45,020,190	42,314,946	81.08	6.35	0.03	97.87	93.83	43.89
CK-2	44,501,442	42,549,490	81.41	6.38	0.03	97.96	94.03	43.78
CK-3	43,678,542	41,507,930	81.48	6.23	0.03	97.94	93.97	43.99
CL_75_-1	47,133,128	46,083,746	82.46	6.91	0.03	98.08	94.34	43.97
CL_75_-2	43,255,808	42,286,636	81.45	6.34	0.03	97.81	93.65	43.82
CL_75_-3	53,348,448	50,859,102	81.84	7.63	0.03	97.77	93.55	43.97

**Table A2:** Basic physicochemical properties of the tested soil.

Soil Type	pH	Conductivity (μs·cm^−1^)	Salt Salinity (g·kg^−1^)	Organic Matter (g·kg^−1^)	Alkali-Hydrolyzed Nitrogen (mg·kg^−1^)	Available Potassium (mg·kg^−1^)	Available Phosphorus (mg·kg^−1^)
loam	8.03	609.43	2.87	21.34	17.08	143.07	22.05
